# Context Matters: Mapping Social Determinants of Health and Physical Function in Older People with HIV Using Geolocation Data

**DOI:** 10.1007/s11524-026-01116-9

**Published:** 2026-07-24

**Authors:** Evelyn Iriarte, Melissa P. Wilson, Pranav Padmanabhan, Joshua A. Barocas, Grace L. Kulik, Allison Webel, Vitor H. F. Oliveira, Vincent Khuu, Paul Cook, Debashis Ghosh, Catherine Jankowski, Kristine M. Erlandson

**Affiliations:** 1 College of Nursing, University of Colorado Anschutz Medical Campus, 13120 E 19Th Ave, Aurora, CO 80045 USA; 2 Department of Medicine, University of Colorado Anschutz Medical Campus, Aurora, CO USA; 3 School of Nursing, University of Washington, Seattle, WA USA; 4 Frances Payne Bolton School of Nursing, Case Western Reserve University, Cleveland, OH USA

**Keywords:** HIV, Older, Determinants of health, Physical function, And Frailty

## Abstract

Older people with HIV (PWH) experience higher rates of physical function decline and frailty than those without HIV. This study aimed to examine the association between physical function measures and geolocated social determinants of health (SDOH) in two geographically distinct groups of older sedentary PWH. We conducted a cross-sectional analysis using baseline data from a randomized exercise trial (the High-Intensity Exercise Study to Attenuate Limitations and Train Habits in Older Adults with HIV; HEALTH) of 120 sedentary PWH aged ≥ 50 years from two sites (Aurora/Denver, Colorado [CO], and Seattle, Washington [WA]). Geolocated SDOH (neighborhood socioeconomic status, mobility infrastructure, environmental amenities and hazards, and access to healthcare) were derived from residential addresses and linked with physical function indicators (400-m walk time, chair rise time, PROMIS function, steps per day, and frailty). Spearman’s correlations were used to quantify the associations between the study variables. For CO, a higher Area Deprivation Index (ADI) was significantly associated with slower chair rise times. In WA, a higher Social Vulnerability Index (SVI) correlated with slower 400-m walk times, and a greater distance to parks was observed among pre-frail/frail participants. Pooled analyses revealed that participants classified as pre-frail/frail resided in neighborhoods with significantly higher SVI scores. Higher neighborhood deprivation, greater social vulnerability, and increased distance to parks were significantly associated with poorer physical function, with site-specific patterns. These findings suggest that the neighborhood context may influence functional health and highlight the need for larger studies to inform place-based interventions.

**Supplementary Information:**

The online version contains supplementary material available at https://doi.org/10.1007/s11524-026-01116-9.

## Introduction

With the advent of effective antiretroviral therapy (ART), life expectancy among people with HIV (PWH) has improved substantially, resulting in an aging population [1]. Currently, 54% of PWH in the U.S. are 50 years old or older [2], and this number is expected to increase to 73% by 2030 [3]. However, aging with HIV infection presents unique challenges. The synergistic effects of HIV infection and aging accelerate the onset and increase the prevalence of age-related conditions, particularly frailty and physical function decline, compared with those without HIV infection [4].

Emerging evidence highlights the critical role of the social determinants of health (SDOH) in shaping aging-related outcomes [5]. SDOH encompass a wide range of contextual factors, including housing, transportation, and neighborhood characteristics that influence health risks and resources [6]. For older PWH who already face elevated risks of physical impairment, adverse SDOH may compound existing vulnerabilities [7–9].

Socioeconomic disadvantages, commonly measured through composite indices such as the Area Deprivation Index (ADI) and Social Vulnerability Index (SVI), have been linked to a range of adverse health outcomes. These indices are derived from census tract-level data and reflect multiple dimensions of socioeconomic conditions. The ADI includes indicators such as income, education, employment, and housing quality [10, 11]. The SVI captures multiple dimensions of community-level stressors, such as socioeconomic status, household composition, and housing and transportation conditions, which can hinder access to health-promoting resources [12]. In the context of HIV, structural disadvantages have been associated with delayed viral suppression [13], suggesting that a place-based disadvantage may directly affect HIV outcomes.

We previously examined the relationship between the ADI and frailty in two urban cohorts of older PWH and found that higher neighborhood deprivation was significantly associated with lower physical activity levels [9]. These findings suggest that specific components of physical function may be more sensitive to contextual socioeconomic factors than to broader frailty classification.

As the PWH population continues to age, understanding how geolocated SDOH influence physical function is essential for promoting healthy aging in this group. This study aimed to examine the cross-sectional association between geolocated SDOH and physical function among two geographically distinct cohorts of sedentary older PWH. We hypothesized that individuals living in neighborhoods characterized by greater socioeconomic disadvantage, fewer environmental amenities (e.g., green spaces, walkable infrastructure), limited healthcare access, and higher environmental hazards (e.g., pollution) will exhibit poorer physical function. Our findings may inform targeted place-based strategies to promote physical health and mitigate structural barriers to healthy aging in this population.

### Theoretical Model

This study is grounded in Bronfenbrenner’s Socio-Ecological Model, which emphasizes the interplay between individuals and their surrounding environments across multiple levels, from immediate interpersonal contexts to broader societal structures [14, 15]. This framework provides a comprehensive approach for understanding how physical function among PWH is influenced not only by individual health behaviors, but also by the layered impact of SDOH. Specifically, factors such as neighborhood deprivation, healthcare access, and environmental amenities and hazards correspond to the model’s exosystem and macrosystem levels, which shape health outcomes through both direct and indirect pathways [14, 15]. Applying this model helps contextualize our findings by illustrating how health is embedded within a web of interrelated systems, and supports the need for multilevel place-based interventions that address upstream determinants to promote healthy aging in older PWH.

## Methods

### Study Design

This study used baseline data from a multisite randomized trial, the High-Intensity Exercise Study to Attenuate Limitations and Train Habits in Older Adults with HIV (HEALTH; ClinicalTrials.gov NCT04550676), conducted at the University of Washington (Seattle, WA, USA) and the University of Colorado Anschutz Medical Campus (Aurora, CO, USA). The parent trial compared high-intensity interval training versus continuous moderate-intensity exercise to assess the effects on physical function, fatigue, and exercise adherence in older adults with HIV [16]. For the present analysis, we employed a cross-sectional design using baseline measures to examine the associations between geolocated social and environmental determinants of health and physical function. The Colorado Multiple Institutional Review Board served as the single review board for this study. All participants provided written informed consent prior to enrollment.

### Sample and Recruitment

All participants were aged 50 years and older, reported being sedentary at baseline (not exercising 2 days/week or more), reported fatigue (≥ 2.0 on either of the first two screening items on the HIV-Related Fatigue Scale), had an HIV-1 RNA level < 200 copies/mL on ART for a minimum of 12 months prior to enrollment, and had a primary address in Colorado (CO) or Washington (WA). Individuals were excluded if they had major contraindications to high-intensity exercise, such as severe mobility limitations, unstable angina, supplemental oxygen use, or uncontrolled hypertension [16].

### Study Variables

Data were collected through a standardized questionnaire using secure web-based software (REDCap) [17], which included participant demographics, HIV-related clinical characteristics, and physical function measures. In addition, geolocated SDOH were derived by linking participants’ residential addresses to publicly available spatial datasets.

### Predictors: Geolocated SDOH Measures

Geospatial predictors were selected a priori based on the published literature and processed using QGIS version 3.30.0. Participant addresses were geocoded into latitude and longitude coordinates and imported as shape files for spatial mapping. External geospatial datasets were obtained in shapefile format whenever possible, or converted from CSV or raster files into shapefiles.

Geolocated SDOH variables were grouped into five domains: (1) Socioeconomic status, including the ADI (national percentile rank from 1 to 100, with higher values indicating greater deprivation) and the SVI (range 0.0 to 1.0, with higher values indicating greater vulnerability); (2) Mobility infrastructure, including Walk Score®, Bike Score®, and Transit Score® (all ranging from 0 to 100, with higher scores reflecting greater walkability, bikeability, or transit access), street connectivity (number of street intersections within a 0.25-mile buffer), and proximity to public transit; (3) Environmental amenities, including proximity to parks and recreation centers, park acreage within a 0.25-mile buffer, park access (binary variable based on a 0.5-mile threshold), and greenness as measured by the Normalized Difference Vegetation Index (NDVI); (4) Environmental hazards, including proximity to highways, pollution levels (PM2.5), vehicular traffic burden (EPA EJScreen indicator), and local crime rates; and (5) Access to healthcare, measured as the Euclidean distance to the nearest Ryan White HIV care facility.

Distance-based variables, such as proximity to the nearest park, recreational facilities, highways, transit stops, and Ryan White HIV healthcare facilities, were calculated using network-based distances derived via the OD-Matrix from the Layers as Lines algorithm in the QNEAT3 plugin within QGIS. Supplemental Table 1 provides a complete list of geospatial variables and data sources.

### Outcomes: Physical Function Measures

Functional outcomes were assessed prior to the intervention and included multiple domains. Time to rise from a chair 10 times and 400-m walk time (400-m walk time), completed as 8 laps on a 25-m course (CO) or 10 laps on a 20-m course (WA), were used as objective indicators of physical function. A subjective measure of physical function, the Form PROMIS Function survey (8b, version 2.0), was also collected prior to the initiation of exercise and scored using its validated algorithm [18]. To assess physical activity, the adult short average steps per day were extracted from an ActiGraph GT3X/1 monitor (ActiGraph, LLC, Fort Walton Beach, FL) worn on the participant’s non-dominant hip for 7–10 consecutive days during the waking hours prior to the initiation of the intervention. Wear time validation was required for at least 10 h per day for a minimum of four consecutive valid days, including at least one weekend day. Frailty status was assessed using the Fried frailty phenotype, with pre-frail and frail classifications combined owing to the low prevalence of frailty [19].

## Statistical Analysis

Participant characteristics, physical function measures, and geolocated SDOH measures were summarized and reported as mean (standard deviation; SD), median (25th, 75th quartile), or count (%) for normally distributed, non-normally distributed, and categorical measures, respectively. Geolocated SDOH were examined by site using t-tests or chi-squared tests for continuous and categorical variables, respectively (non-parametric or exact alternatives were employed, where appropriate).

Associations between frailty (frail or pre-frail vs. non-frail) and geolocated SDOH were explored using t-tests or chi-square tests (or non-parametric alternatives). Due to study site differences, associations between continuous measures of physical function and geolocated SDOH were explored using Spearman correlations stratified by site. As we found marked site-specific differences in the distributions of several physical function and geolocated variables, pooled analyses risked generating misleading or contradictory associations (Supplemental Fig. 1). Therefore, we report and discuss primarily the site-specific findings and only provide pooled analyses for our primary frailty outcome.

P-values for Spearman correlations were approximated by assuming a t-distribution [20]. Statistical significance was set at 0.05, with no adjustment for multiple comparisons. All analyses were performed using the RStudio software (version 4.4.3, Vienna, Austria).

## Results

Of the 142 participants who consented to be screened in the parent study, 120 had complete residential addresses and physical function data and were included in this analysis. The demographic and clinical characteristics of the participants are presented in Table 1. Participants in the CO and WA groups differed significantly in multiple geolocated SDOH (Table 2). Compared to WA, participants in CO lived in neighborhoods with higher ADI scores; lower walk and transit scores; greater distances to highways, transit, and parks; and lower average greenness. CO participants also had faster average 400-m walk times and chair rise times, higher daily step counts, and were more likely to score a point for weakness on frailty evaluation. Over half of the total sample (54.2%) was classified as pre-frail or frail based on the Fried criteria, with 27.5% reporting low physical activity and 28.3% reporting exhaustion.

**Table 1:** Demographic and clinical characteristics of participants in CO and WA study sites (n = 120)

	Colorado (*n* = 60)	Washington (*n* = 60)	P-value
Age—median [IQR]	58.0 [54.0, 61.3]	56.0 [53.0, 61.3]	0.63^a^
Sex – n (% female)	9 (15.0%)	7 (12.0%)	0.79
Race/Ethnicity—n (%)			
African American	11 (18.3%)	9 (15.0%)	0.15^b^
Hispanic (regardless of race)	12 (20.0%)	5 (8.3%)	
White/Anglo, non-Hispanic	35 (58.3%)	40 (66.7%)	
Other	2 (3.3%)	6 (10.0%)	
Education – n (%)			0.93
< 11 years, high school or GED	11 (18.3%)	10 (16.7%)	
Some college	22 (36.7%)	24 (40.0%)	
Completed college, bachelor's degree, or post-college	27 (45.0%)	26 (43.3%)	
Employment – n (%)			0.27
Disabled (permanent or temporary)	11 (18.3%)	16 (26.7%)	
Retired	15 (25.0%)	10 (16.7%)	
Work part-time or full-time	28 (46.7%)	23 (38.3%)	
Other/Missing	6 (10.0%)	11 (18.3%)	
Current smoker – n (%)	6 (10.0%)	8 (13.3%)	0.78
Illicit drug use in the last 2 years – n (% yes)	5 (8.3%)	11 (18.3%)	0.18
Years since HIV diagnosis—median [IQR]	22.5 [15.0, 29.3]	24.0 [19.8, 30.3]	0.21^a^
Years of ART—median [IQR]	19.5 [12.8, 27.0]	19.5 [12.8, 25.3]	0.68^a^
Current CD4 T-cell count – mean (SD)	770.4 (353.3)	638.53 (237.5)	0.02
Body mass index– mean (SD)	30.5 (6.0)	29.3 (5.7)	0.26
Hypertension – n (%)	34 (56.7%)	27 (45.0%)	0.27
Hyperlipidemia – n (%)	30 (50.0%)	36 (60.0%)	0.36
Depression, anxiety, or bipolar disorder – n (%)	29 (48.3%)	37 (61.7%)	0.20
Diabetes – n (%)	7 (11.7%)	13 (21.7%)	0.21
Cardiovascular disease – n (%)	4 (6.7%)	5 (8.3%)	1.0
VACS score – mean (SD)	32.3 (9.3)	33.0 (9.3)	0.69

ART = antiretroviral therapy; SD = standard deviation; VACS = Veterans Aging Cohort Study (VACS) 2.0 Index. ^a^non-parametric testing; ^b^exact version of test was performed

**Table 2:** Physical function and geolocated SDOH

	Overall (*n* = 120)	Colorado (*n* = 60)	Washington (*n* = 60)	p-value
Physical function measures				
400- m walk time (sec)^a^ – median [IQR]^b^	261.6 [245.4, 283.1]	251.4 [238.3, 277.8]	272.2 [254.1, 290.1]	0.007
Time to rise from chair (sec) – mean (SD)	21.1 (5.6)	19.7 (4.9)	22.5 (6.0)	0.006
Average steps per day (actigraph) – median [IQR]^b^	5992.2 [4751.1, 11044.8]	10444.9 [5670.2, 12970.6]	5303.0 [3812.9, 6857.3]	< 0.001
PROMIS function T-score – median [IQR]^b^	49.2 [43.1, 60.1]	47.8 [43.1, 56.5]	49.2 [43.7, 60.1]	0.661
Frailty				
Fried frailty category – n (% pre-frail or frail)	65 (54.2)	36 (60.0)	29 (48.3)	0.272
Frailty points – median [IQR]^b^	1.0 [0.0, 1.0]	1.0 [0.0, 2.0]	0.0 [0.0, 1.0]	0.141
Weakness – n (% yes)	22 (18.3)	16 (26.7)	6 (10.0)	0.034
Slowness – n (% yes)	0 (0.0)	0 (0.0)	0 (0.0)	NA
Weight loss – n (% yes)^c^	7 (5.8)	1 (1.7)	6 (10.0)	0.114
Low activity – n (% yes)	33 (27.5)	18 (30.0)	15 (25.0)	0.683
Exhaustion – n (% yes)	34 (28.3)	20 (33.3)	14 (23.3)	0.311
Socioeconomic status (SES)				
ADI national – median [IQR]^b^	18.0 [9.0, 30.0]	29.0 [18.0, 39.0]	11.0 [6.0, 17.0]	< 0.001
SVI percentile – median [IQR]^b^	60.6 [38.9, 85.1]	65.7 [42.2, 86.6]	57.5 [34.7, 83.6]	0.527
Mobility				
Bike score – median [IQR]^b^	67.0 [51.0, 85.0]	61.0 [53.0, 78.0]	73.0 [48.8, 88.0]	0.295
Walk score – median [IQR]^b^	68.0 [48.0, 90.0]	57.0 [40.0, 73.5]	80.0 [60.5, 96.0]	< 0.001
Transit score – median [IQR]^b^	50.0 [38.0, 64.5]	40.5 [35.3, 50.8]	60.0 [47.0, 76.0]	< 0.001
Street connectivity – median [IQR]^b^	106.0 [72.0, 140.0]	100.0 [72.0, 132.0]	113.0 [70.5, 142.0]	0.462
Proximity to public transit (mi) – median [IQR]^b^	0.1 [0.0, 0.2]	0.1 [0.1, 0.2]	0.1 [0.03, 0.10]	< 0.001
Environmental amenities				
NDVI – mean (SD)	0.4 (0.2)	0.4 (0.1)	0.5 (0.2)	0.012
Distance to nearest park (mi) – median [IQR]^b^	0.2 [0.1, 0.3]	0.3 [0.2, 0.3]	0.2 [0.1, 0.3]	0.014
Park acreage area with 0.25 miles (sqkm) – median [IQR]^b^	0.01 [0.00, 0.04]	0.02 [0.0, 0.1]	0.01 [0.0, 0.03]	0.186
Park access (within 0.5 miles) – n (% no)	27 (26.2)	16 (37.2)	11 (18.3)	0.055
Recreation facilities (within 0.5 miles) – n (% yes)	20 (23.5)	9 (20.9)	11 (26.2)	0.752
Environmental hazards				
Pollution levels (particulate matter 2.5 level) – median [IQR]^b^	9.1 [8.9, 9.2]	9.0 [8.8, 9.3]	9.1 [9.0, 9.2]	0.148
Proximity to highway – median [IQR]^b^	1.0 [0.5, 1.6]	1.3 [0.8, 2.3]	0.7 [0.3, 1.2]	0.001
Vehicular traffic – median [IQR]^b^	3105524.7 [2022507.7, 4521068.9]	2896669.4 [2016487.2, 4535178.8]	3126785.6 [2072345.3, 4521068.9]	0.517
Crime rate 2022 – median [IQR]^b^	108.3 [50.3, 207.1]	125.6 [52.6, 302.6]	107.5 [50.3, 150.3]	0.438
Access to healthcare				
Proximity to HIV clinics and hospitals – median [IQR]^b^	3.3 [1.2, 6.9]	3.6 [1.2, 6.9]	1.9 [1.0, 6.9]	0.271

ADI = Area Deprivation Index; IQR = interquartile range; NDVI = Normalized Difference Vegetation Index; SD = standard deviation; SVI = Social Vulnerability Index

^a^One participant was unable to complete the 400-m walk time due to severe hip pain

^b^Non-parametric test

^c^Exact test

## Site-Specific Associations Between Physical Function and Geolocated SDOH

**Fig. 1:**
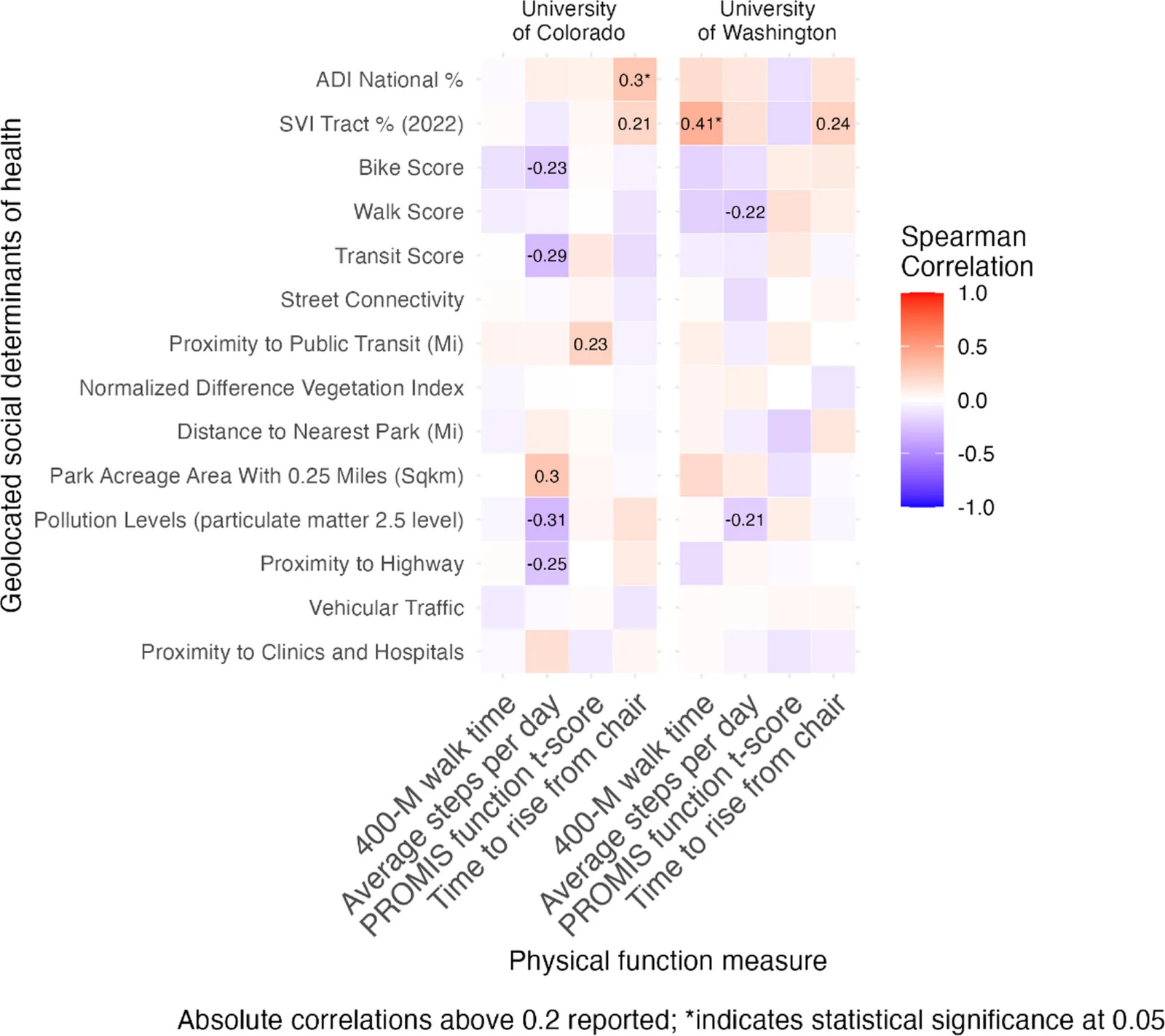
Correlations between physical function and geolocated SDOH by study site

To account for between-site heterogeneity, we examined the correlations between physical function outcomes and SDOH, separately by site. For CO, higher ADI scores were associated with a longer time to complete the chair rise test (ρ = 0.30, *p* < 0.05). In WA, higher SVI scores were associated with slower 400-m walking times (ρ = 0.41, *p* < 0.05). Additional associations are illustrated in Fig. 1; however, they did not reach statistical significance.

## Site-Stratified and Pooled Associations Between Frailty and Geolocated SDOH

We next stratified the participants by frailty status (pre-frail or frail vs. non-frail). In CO, pre-frail/frail participants tended to live in neighborhoods with higher SVI (74.8 vs. 52.9, *p* = 0.097) and ADI scores (31.0 vs. 24.0, *p* = 0.14), though these differences were not statistically significant (Supplemental Table 2). In Washington, pre-frail or frail participants lived farther from parks (0.22 vs. 0.15 miles; *p* = 0.016). (*p* = 0.016) and tended to live in neighborhoods with higher SVI scores (*p* = 0.061) and lower bike scores (*p* = 0.062) than non-frail participants.

Lastly, we explored the univariate associations between frailty status and geolocated SDOH in pooled analyses (Table 3). Participants classified as pre-frail or frail lived in census tracts with higher SVI scores, indicating greater vulnerability (73.9 vs. 54.4, *p* = 0.01). Although not statistically significant, similar trends were observed for higher ADI scores, greater distances to the nearest park, and increased distances to HIV clinics and hospitals (Table 4).

**Table 3:** Pooled univariable associations between frailty and geolocated SDOH

	Overall (*n* = 120)	Non-frail (*n* = 55)	Pre-frail or frail (*n* = 65)	p-value
Socioeconomic status (SES)				
ADI national – median [IQR]^a^	18.0 [9.0, 30.0]	15.0 [7.0, 28.0]	21 [11.0, 33.0]	0.062
SVI percentile – median [IQR]^a^	60.6 [39.0, 85.1]	54.4 [24.8, 81.5]	73.9 [52.3, 86.6]	0.01
Mobility				
Bike score – median [IQR]^a^	67.0 [51.0, 85.0]	75.0 [56.0, 88.5]	63 [49.3, 80.8]	0.064
Walk score – median [IQR]^a^	68.0 [48.0, 90.0]	74.0 [57.0, 92.5]	63.5 [43.3, 79.3]	0.057
Transit score – median [IQR]^a^	50.0 [38.0, 64.5]	51.0 [43.5, 67.5]	42.0 [36.0, 64.0]	0.083
Street connectivity – median [IQR]^a^	106.0 [72.0, 140.0]	100.0 [65.0, 141.0]	108.0 [73.5, 140.0]	0.685
Proximity to public transit (mi) – median [IQR]^a^	0.1 [0.0, 0.2]	0.1 [0.04, 0.1]	0.1 [0.1, 0.2]	0.492
Environmental amenities				
NDVI – mean (SD)	0.4 (0.2)	0.5 (0.2)	0.4 (0.1)	0.74
Distance to nearest park (mi) – median [IQR]^a^	0.2 [0.1, 0.3]	0.2 [0.1, 0.3]	0.2 [0.2, 0.3]	0.089
Park acreage area with 0.25 miles (sqkm) – median [IQR]^a^	0.01 [0.00, 0.04]	0.01 [0.00, 0.02]	0.01 [0.00, 0.06]	0.43
Park access (within 0.5 miles) – n (% no)	27 (26.2)	11 (22.0)	16 (30.2)	0.471
Recreation facilities (within 0.5 miles) – n (% yes)	20 (23.5)	7 (17.1)	13 (29.5)	0.272
Environmental hazards				
Pollution levels (particulate matter 2.5 level) – median [IQR]^a^	9.1 [8.9, 9.2]	9.1 [9.0, 9.3]	9.0 [8.9, 9.2]	0.182
Proximity to highway – median [IQR]^a^	1.0 [0.5, 1.6]	1.0 [0.4, 1.6]	1.0 [0.5, 1.7]	0.827
Vehicular traffic – median [IQR]^a^	3105524.7 [2022507.7, 4521068.9]	3212090.6 [1927687.6, 4521068.9]	3022024.64 [2051325.3, 4406108.7]	0.668
Crime rate 2022 – median [IQR]^a^	108.3 [50.3, 207.1]	108.3 [51.3, 177.1]	106.0 [49.8, 207.8]	0.66
Access to healthcare				
Proximity to HIV clinics and hospitals – median [IQR]^a^	3.3 [1.2, 6.9]	1.9 [0.9, 6.4]	3.8 [1.4, 7.0]	0.057

ADI = Area Deprivation Index; IQR = interquartile range; NDVI = Normalized Difference Vegetation Index; SD = standard deviation; SVI = Social Vulnerability Index

^a^Non-parametric test

**Table 4:** Multivariable model results

Model (dependent variable & primary SDOH independent variable)	Chair Rise ~ ADI Adj. Estimate [95%CI]	Chair Rise ~ SVI Adj. Estimate [95%CI]	400-MWT ~ SVI Adj. Estimate [95%CI]	Frailty ~ SVI Adj OR [95%CI]
Age (per 10 years)	0.30 [−1.43, 1.93]; 0.769	0.58 [−1.03, 218]; 0.477	20.1 [8.20, 31.98]; 0.001*	1.28 [0.68, 2.47]; 0.446
Female birth sex	3.45 [0.57, 6.34]; 0.019*	3.43 [0.63, 6.23]; 0.017*	55.11 [34.37, 75.85]; < 0.001*	1.68 [0.55, 5.51]; 0.370
Site: Washington	4.35 [1.98, 6.72]; < 0.001*	3.10 [1.18, 5.01]; 0.002*		0.69 [0.32, 1.47]; 0.340
SDOH (per 10%-points; no interaction)	0.86 [0.07, 1.66]; 0.034*	4.82 [1.37, 8.28]; 0.007*		1.22 [1.07, 1.42]; 0.005*
SDOH – Washington			59.10 [24.14, 94.04]; 0.001*	
SDOH—Colorado			3.92 [−33.51, 41.35]; 0.836	

ADI = Area Deprivation Index; KNN = k-nearest neighbors; SDOH = social determinant of health; SVI = Social Vulnerability Index

^*^indicates significance at 0.05. Testing of residuals for spatial correlation via Moran’s I tests returned non-significant results for all models (P > 0.69)

## Discussion

This study examined the association between physical function indicators and geolocated SDOH among older PWH across two distinct U.S. regions. While research on this topic remains limited, particularly in the context of aging with HIV, emerging evidence suggests that socioeconomic disadvantage may play a critical role in shaping frailty vulnerability and physical function outcomes [7–9].

In our study, we found that higher neighborhood-level social vulnerability was associated with poorer physical function, including slower gait speed (WA) and increased frailty (pooled analyses). The SVI captures multiple dimensions of community-level stressors, such as socioeconomic status, household composition, and housing and transportation conditions, which can hinder access to health-promoting resources (Agency for Toxic Substances and Disease Registry, 2024). These findings align with previous research linking higher SVI to increased rates of HIV diagnosis and adverse health outcomes [21], and underscore the relevance of addressing structural vulnerability in efforts to promote healthy aging.

Similar trends have been observed in studies of older adults without HIV, where disadvantaged neighborhood environments are linked to worse physical function [22]. This association is likely multifactorial; higher social vulnerability may limit access to quality healthcare, healthy food, safe housing, and opportunities for physical activity [23]. Higher vulnerability is also associated with increased psychosocial stress, depression, and anxiety, which can further impair physical function [24]. Therefore, interventions targeting upstream SDOH may be crucial for enhancing physical health and quality of life among older PWH.

Similar to SVI, we found that greater socioeconomic deprivation, captured through the ADI, was associated with slower chair rise time, a key indicator of lower limb strength, among participants in CO. Previous research has linked higher ADI scores to poorer health outcomes in other populations; among older adults without HIV, ADI has been associated with an increased risk of chronic disease, COVID-19 mortality, hospital readmissions, and Alzheimer’s disease [25–28]. In PWH, a higher ADI predicted greater mortality among women with HIV [29], brain aging [30], and lower physical activity [9]. In contrast, another study found that a higher ADI was linked to a lower HIV/AIDS risk among U.S. Veterans [31]. These mixed results highlight the complexity of neighborhood disadvantage and its varied impact on health in PWH.

Our results also support the relevance of built environment features, such as access to green spaces and infrastructure for active transportation, in supporting physical function. Participants who lived farther from parks or in neighborhoods with lower bike scores were more likely to be pre-frail or frail, particularly in WA. These findings mirror studies in older adults showing positive effects of walkability and park access on physical activity and mobility [32, 33]. Although research on this topic remains limited in PWH, built environment factors have been linked to HIV care engagement and healthcare utilization [34–36]. Our findings extend this work by suggesting a potential link between neighborhood design and functional decline. Community-level interventions that enhance mobility, green space access, and neighborhood safety may help delay frailty onset and support healthy aging.

Site-level differences further highlight the influence of place. Participants in the CO site had slower gait speed, more daily steps, and lived in neighborhoods with higher deprivation and lower walkability and transit scores. These geographic differences underscore the importance of tailoring interventions in the local context. Prior work has shown that “place” has a profound influence on health outcomes, particularly among marginalized groups [34]. Strategies that overlook neighborhood-specific barriers, such as transportation gaps or park access, may fail to improve functional health in older PWH [35]. Our findings support the need for place-based, community-informed approaches to reduce age-related challenges in this population.

The site-level differences found in our study also raise the possibility of threshold effects, in which environmental factors may begin to influence physical function only after crossing certain levels of deprivation or support. This perspective suggests that contextual factors may have a limited impact until the basic threshold is unmet. Future studies could explore this potential nonlinear relationship using analytic approaches, such as classification trees, to identify inflection points across diverse settings.

## Limitations

Given the cross-sectional nature of our data, causal inferences could not be made. In addition, there may still be unmeasured confounding, both spatial and non-spatial, that needs to be accounted for in more formal modeling. The use of a convenience sample from two existing study sites in CO and WA limits the generalizability of our findings, as participants enrolled in clinical research may differ from the broader population, potentially having lower social vulnerability due to greater health engagement or access to resources. Despite this potential bias, the observed associations suggest that the relationship between social vulnerability and physical function outcomes may be stronger in the general population. Our results highlight that the demographic, social, and environmental determinants of PWH health profiles can vary considerably across different regions of the United States. The sample size was relatively small, and some non-significant trends may reflect insufficient statistical power. Future studies with larger sample sizes and longitudinal designs are necessary to validate these findings and to explore potential mechanisms.

## Conclusions

In our sample of older PWH, higher neighborhood deprivation, greater social vulnerability, and increased distance to parks were significantly associated with poorer physical function outcomes, with distinct patterns observed across the study sites. While these associations were modest in strength, they point to the potential influence of neighborhood-level disadvantage on functional health among older PWH. These preliminary findings underscore the need for larger, longitudinal studies to confirm these associations and inform targeted place-based interventions that promote functional health and healthy aging in this population.

## Supplementary Information

Below is the link to the electronic supplementary material.Supplementary file1 (DOCX 20 KB)Supplementary file2 (DOCX 17 KB)Supplementary file3 (DOCX 21 KB)
