# Supplementary material for: Context Matters: Mapping Social Determinants of Health and Physical Function in Older People with HIV Using Geolocation Data
Source: J Urban Health. 2026 Jul 24;103(4):872–82. doi: 10.1007/s11524-026-01116-9 (PMC13524893; doi:10.1007/s11524-026-01116-9)
Supplement: Supplementary file 1 [file 11524_2026_1116_MOESM1_ESM.docx]

**Supplemental Table 1**

*Independent Variables Describing Geolocated Social Determinants of Health*

| **Variable name** | **Variable definition** | **Data source(s)** |
| --- | --- | --- |
| Address | Participant's address of residence | HEALTH study |
| Latitude | Geocoded latitude of participant residence (in degrees) | HEALTH study |
| Longitude | Geocoded longitude of participant residence (in degrees) | HEALTH study |
| **Socio-economic status** |  |  |
| Area Deprivation Index (ADI) national^a^ | National rank of ADI in 2022 of the census block group in which the participant resides. Range: 1 to 100 (national percentile rankings). Higher ADI scores = Greater socioeconomic disadvantage. | University of Wisconsin Center for Health Disparities Research Neighborhood Atlas |
| Social Vulnerability Index (SVI) | SVI score of the census tract in which the participant resides. Range: 0–100 (percentile). Higher SVI scores = Greater vulnerability. | U.S. CDC Agency for Toxic Substances and Disease Registry |
| **Mobility** |  |  |
| Bike score | Bike score calculated at the participant's residence. Range: 0 to 100. Higher scores indicate more favorable conditions (more walkable). | Walk Score database Walkscore.com |
| Walk score^b^ | Walk score calculated at the participant's residence. Range: 0 to 100. Higher scores indicate more favorable conditions (more bikeable). | Walk Score database Walkscore.com |
| Transit score^c^ | Transit score calculated at the participant's residence. Range: 0 to 100. Higher scores indicate better transit access. | Walk Score database Walkscore.com |
| Street connectivity | Number of street intersections within a 0.25-mile Euclidean buffer from the participant's address | U.S. Census Bureau TIGER/Line shapefiles, 2023, by county |
| Proximity to public transit | Euclidean distance (in miles) from participant address to nearest transit system stop (RTD in Colorado; King County Metro in Washington) | 1. Colorado Regional Transportation District (RTD) 2. King County (WA) Metro |
| **Environmental Amenities** |  |  |
| Normalized Difference Vegetation Index (NDVI) | Raw NDVI raster value at participant address | United States Geological Survey (USGS) eMODIS NDVI Raster |
| Proximity to nearest park | Euclidean distance (in miles) from participant address to nearest park centroid | 1. City of Denver 2. City of Aurora 3. King County, WA 4. Snohomish County, WA |
| Park acreage area^d^ | Area (in square kilometers) of parks within a 0.25-mile Euclidean buffer of participant address | 1. City of Denver 2. City of Aurora 3. King County, WA   Snohomish County, WA |
| Park access^d^ | Binary indicator of whether the network distance from the participant's address to the nearest park centroid is <0.5 miles | 1. City of Denver 2. City of Aurora 3. King County, WA 4. Snohomish County, WA |
| Recreation facilities^e^ | Binary indicator of whether Euclidean distance from participant address to nearest recreation facility is <0.5 miles | 1. City of Denver 2. City of Aurora 3. City of Seattle |
| **Environmental Hazards** |  |  |
| Pollution levels | Annual average um/m^3^ of PM _2.5_ (particulate matter) within the census block group in which the participant resides | U.S. Environmental Protection Agency Environmental Justice Screen |
| Proximity to highway | Euclidean distance (in miles) from participant's address to the nearest point on a major primary highway | U.S. Census Bureau TIGER/Line shapefiles, 2023, by state |
| Vehicular traffic | EJScreen Traffic Proximity Indicator (the count of vehicles per day (average annual daily traffic- AADT) divided by distance) within the census block group in which the participant resides | U.S. Environmental Protection Agency Environmental Justice Screen |
| Crime rate^f^ | Number of recorded total crimes in 2022 per 1,000 residents within the census block group in which the participant resides | 1. Denver Police Department 2. Seattle Police Department 3. U.S. Census Bureau 4. ACS 5-year 2021 |
| **Access to Healthcare** |  |  |
| Proximity to HIV clinics and hospitals | Euclidean distance (in miles) from participant's address to the nearest Ryan White HIV healthcare facility | U.S. Health Resources and Services Administration (HRSA) |

**Notes.**

a. Missing values: 21. CBGs with suppressed values of ADI (“GQ”) were excluded.
b. Missing values: 16.
c. Missing values: 2.
d. Missing values: 19. CO participants outside of Denver or Aurora excluded due to data unavailability.
e. Missing values: 41. Participants outside Denver, CO, Aurora, CO, or Seattle, WA, were excluded due to data unavailability.
f. Missing values: 70. Participants outside Denver, CO, or Seattle, WA, were excluded due to data unavailability.
