# Supplementary material for: Context Matters: Mapping Social Determinants of Health and Physical Function in Older People with HIV Using Geolocation Data
Source: J Urban Health. 2026 Jul 24;103(4):872–82. doi: 10.1007/s11524-026-01116-9 (PMC13524893; doi:10.1007/s11524-026-01116-9)
Supplement: Supplementary file 2 [file 11524_2026_1116_MOESM2_ESM.docx]

**Supplemental Table 2**

*Spearman Correlation and Lee’s L Spatial Correlation For Significant Site-Specific Associations*

| **Physical Function Measure** | **SDOH Neighborhood Measure** | **Spearman Correlation** | **Lee’s L Spatial Correlation Statistic; p-value** | | | |
| --- | --- | --- | --- | --- | --- | --- |
|  |  |  | **Min-Max** | **KNN=5** | **KNN = 6** | **KNN=7** |
| **University of Colorado** | | | | | | |
| Chair Rise 10x | ADI | 0.30* | 0.01;  0.985 | 0.13;  **0.050*** | 0.11;  0.106 | 0.10;  0.086 |
| Chair Rise 10x | SVI | 0.21 | -0.00;  0.707 | 0.05;  0.761 | 0.03;  0.929 | 0.02;  0.725 |
| **University of Washington** | | | |  |  |  |
| Chair Rise 10x | SVI | 0.24 | 0.06;  0.164 | 0.10;  0.146 | 0.08;  0.186 | 0.09;  0.058 |
| 400-Meter walk time | SVI | 0.41* | 0.12;  **0.010*** | 0.08;  0.461 | 0.08;  0.440 | 0.08;  0.444 |

**Note.** ADI= Area Deprivation Index; KNN= k-nearest neighbors; SDOH= social determinant of health; SVI= Social Vulnerability Index.

* indicates significance at 0.05; p-values for Lee’s L calculated via permutation tests over 1000 permutations.

Neighbors used in spatial analyses were identified based on Great Circle distances using latitude and longitude. As 5 participant pairs had identical information, a small amount of random noise was added (U~ +/- 0.00001) to latitudes of one of each pair to facilitate assessments. A min-max critical distance threshold was used to identify neighbors; analyses were stratified by site, and this process was performed for the cohort at each site. The primarily urban setting resulted in a high number of average links (UC: average of 48.5 links for 59 participants; UW: average of 25 links for 60 participants); due to the potential for over-smoothing (loss of locality), a secondary approach limiting neighbors to the 6 nearest was also used in the calculation of Lee’s L statistic. Statistics using KNN 5 and 7 were calculated to provide a sense of robustness of estimates across different thresholds. All results are presented for transparency.

The KNN=6 method was used to construct neighbor definitions pooling both sites and used in the Moran’s I tests for spatial correlation in model residuals.
