# Supplementary material for: Context Matters: Mapping Social Determinants of Health and Physical Function in Older People with HIV Using Geolocation Data
Source: J Urban Health. 2026 Jul 24;103(4):872–82. doi: 10.1007/s11524-026-01116-9 (PMC13524893; doi:10.1007/s11524-026-01116-9)
Supplement: Supplementary file 3 [file 11524_2026_1116_MOESM3_ESM.docx]

**Supplemental Table 2**

*Associations between Frailty and Geolocated Social Determinants of Health by Study Site*

|  | **Colorado** | | | | **Washington** | | |
| --- | --- | --- | --- | --- | --- | --- | --- |
| **Colorado site** | | **Non-frail**  **(*n*=24)** | **Pre-frail or frail (*n*=36)** | **p-value** | **Non-frail**  **(*n*=31)** | **Pre-frail or frail (*n*=29)** | **p-value** |
| **Socioeconomic status (SES)** | |  |  |  |  |  |  |
| ADI national – median [IQR]^a^ | | 24.0 [14.0, 33.5] | 31.0 [22.8, 40.0] | 0.14 | 9.0 [5.0, 16.0] | 12.0 [6.0, 19.0] | 0.445 |
| SVI – median [IQR]^a^ | | 52.9 [27.6, 82.5] | 74.8 [51.8, 86.6] | 0.097 | 54.5 [24.8, 81.5] | 72.9 [53.6, 84.7] | 0.061 |
| **Mobility** | |  |  |  |  |  |  |
| Bike score – median [IQR]^a^ | | 65.5 [44.8, 80.5] | 60.0 [53.0, 74.0] | 0.73 | 80.0 [62.5, 89.0] | 65.0 [37.0, 83.0] | 0.062 |
| Walk score – median [IQR]^a^ | | 59.0 [41.8, 71.5] | 55.0 [39.0, 73.5] | 0.58 | 82.0 [71.0, 96.0] | 77.0 [55.0, 93.0] | 0.188 |
| Transit score – median [IQR]^a^ | | 48.0 [38.0, 51.0] | 38.0 [35.0, 49.0] | 0.15 | 61.0 [50.0, 80.3] | 60.0 [42.0, 74.0] | 0.554 |
| Street connectivity – median [IQR]^a^ | | 99.0 [70.5, 143.5] | 106.0 [74.0, 128.0] | 0.982 | 108.0 [64.0, 138.0] | 120.00 [74.0, 146.0] | 0.429 |
| Proximity to public transit (mi) – median [IQR]^a^ | | 0.1 [0.1, 0.2] | 0.1 [0.1, 0.2] | 0.963 | 0.1 [0.0, 0.1] | 0.1 [0.1, 0.1] | 0.429 |
| **Environmental amenities** | |  |  |  |  |  |  |
| NDVI – mean (SD) | | 0.4 (0.1) | 0.4 (0.1) | 0.788 | 0.5 (0.2) | 0.5 (0.2) | 0.916 |
| Distance to nearest park (mi) – median [IQR]^a^ | | 0.29 [0.21, 0.32] | 0.23 [0.19, 0.32] | 0.420 | 0.15 [0.08, 0.24] | 0.22 [0.17, 0.36] | 0.016 |
| Park acreage area with 0.25 miles (sqkm) – median [IQR]^a^ | | 0.02 [0.00, 0.03] | 0.02 [0.00, 0.07] | 0.267 | 0.01 [0.00, 0.02] | 0.01 [0.00, 0.04] | 0.988 |
| Park access (within 0.5 miles) – n (% no) | | 8 (42.1) | 8 (33.3) | 0.785 | 3 (9.7) | 8 (27.6) | 0.145 |
| Recreation facilities (within 0.5 miles) – n (% yes) | | 3 (15.8) | 6 (25.0) | 0.719 | 4 (18.2) | 7 (35.0) | 0.375 |
| **Environmental hazards** | |  |  |  |  |  |  |
| Pollution levels (particulate matter 2.5 level) – median [IQR]^a^ | | 9.1 [8.8, 9.4] | 9.0 [8.9, 9.3] | 0.752 | 9.2 [9.1, 9.2] | 9.1 [9.0, 9.1] | 0.099 |
| Proximity to highway – median [IQR]^a^ | | 1.3 [0.7, 2.3] | 1.2 [0.8, 2.3] | 0.902 | 0.9 [0.3, 1.3] | 0.7 [0.3, 1.2] | 0.739 |
| Vehicular traffic – median [IQR]^a^ | | 3089292.7 [1740935.4, 4523245.5] | 2819039.4 [2051325.3, 4390205.6] | 0.621 | 3212090.6 [21255878.0, 4521068.9] | 3105524.7 [2098563.7, 4367788.6] | 0.929 |
| Crime rate 2022 – median [IQR]^a^ | | 100.6 [52.7, 210.4] | 130.7 [49.0, 398.9] | 0.908 | 108.3 [54.0, 148.7] | 86.1 [49.8, 157.9] | 0.440 |
| **Access to healthcare** | |  |  |  |  |  |  |
| Proximity to HIV clinics and hospitals – median [IQR]^a^ | | 3.0 [1.0, 7.0] | 3.8 [2.2, 6.9] | 0.347 | 1.7 [0.9, 5.2] | 3.3 [1.2, 7.8] | 0.181 |

*Note.* ADI= Area Deprivation Index; IQR= interquartile range; NDVI= Normalized Difference Vegetation Index; SD= standard deviation; SVI= Social Vulnerability Index.

^a^ Non-parametric test.
